# Vascular bifurcation influences the protein corona composition on nanoparticles and impacts their cellular uptake

**DOI:** 10.1039/d2na00066k

**Published:** 2022-05-13

**Authors:** Sridevi B. Conjeevaram, Ryan M. Blanchard, Amulya Kadaba, Isaac M. Adjei

**Affiliations:** Department of Biomedical Engineering, Texas A&M University College Station TX 77843 USA adjeii@tamu.edu

## Abstract

The protein corona (PC) that forms on nanoparticles (NPs) after *in vivo* injection influences their biodistribution, pharmacokinetics, and cell interaction. Although injected NPs traverse vascular networks, the impact of vascular features on the protein corona composition is mainly unexplored. Using an *in vitro* flow model that introduces bifurcations, a common feature of blood vessels, we show that vessels are not passive bystanders in the formation of the PC but that their features play active roles in defining the PC on NPs. The addition of bifurcation significantly increased the amount of proteins associated with NP. The bifurcation's introduction also changed the PC's composition on the NPs and affected the NP interactions with cells. Correlation analysis and modeling showed that these changes in the PC are mediated by both the branching and diameter reduction associated with vessel bifurcation and the resulting change in flow rate. The results indicate that blood vessel structures play an active part in the information of the PC, and their role should be studied critically for a better understanding of the PC and its biological implications.

## Abstract 2

The article uses *in vitro* models to show blood vessel influence on the protein corona’s (PC) composition. These results highlight the need to investigate the blood vessel architecture’s influence on the PC and their biological fate.

## Graphical abstract

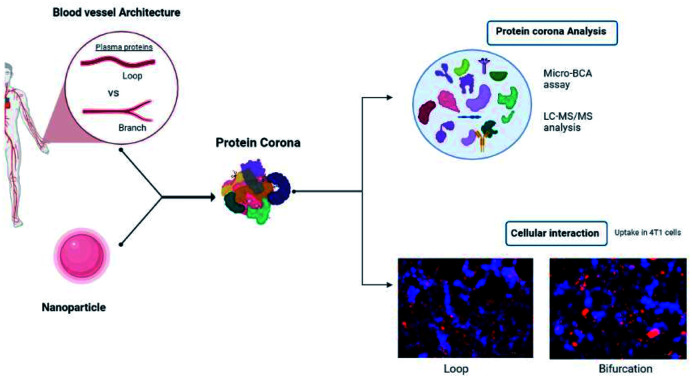

## Introduction

Nanoparticles (NPs) continue to see increasing use in medicine for drug delivery, medical imaging, and diagnosis.^1,2^ NPs have tuneable properties such as size, shape, and surface chemistry that can be controlled for different medical applications.^3^ Their large surface-to-volume ratio allows conjugation of ligands, thereby enhancing their delivery to target tissues for therapy and imaging.^4,5^

After *in vivo* administration into biological fluids, proteins rapidly adsorb on NPs, forming a protein corona (PC) due to their high surface free energy.^6,7^ The PC imparts a new biological identity to the NPs that governs their biological fate. The PC composition influences NP uptake by immune cells with implications for their pharmacokinetics and biodistribution.^8^ It also affects cellular responses, including inflammation, oxidative stress, and apoptosis.^9–11^

The biological effect of the PC on NP fate is determined by the type and amount of the bound proteins, predicted by the NP physicochemical characteristics and the physiological conditions under which the PC is formed.^12,13^ Physicochemical properties of NPs such as size, shape, surface charge, and surface functionalization directly mediate the formation of the PC and influence the composition of the protein corona.^8,12,14^ However, even with extensive studies, there remains discordance between the *in vitro* identified protein corona and those identified on NPs collected from *in vivo* studies for particles with the same physicochemical characteristics.^15^

Recently, the effect of flow on PC composition and its ultimate impact on NP fate has been recognized,^16,17^ with the flow velocity directly affecting the composition of the PC.^13^ These results also demonstrate that shear stress generated under dynamic flow conditions affects the complexity and abundance of protein corona on NPs.^18^ Studies comparing PC formed on NPs under dynamic flow and static conditions *in vitro* show variation in the amount and composition of the PC that affects NPs' cellular uptake and toxicity.^17^

Characterizing the PC formed on intravenously injected NPs *in vivo* and comparing it to PC formed *in vitro* under flow conditions has further highlighted how the complex physiological environment influences the composition of the PC and the resulting biological effect, independent of their physicochemical properties.^19^ Understanding how physiological attributes of the vasculature other than flow velocity influence the composition of the PC is necessary to reconcile the seeming disparity between PCs formed *in vivo* and *in vitro*. Resolution of this problem will enable better prediction of the PC composition on NPs and their potential biological effects.

Bifurcation is a common feature of blood vessels, with larger vessels progressively branching into smaller daughter vessels.^20^ Such changes often lead to variations in blood flow. Poiseuille's law states that the volume flow rate directly depends on the vessel's geometry, such as radius of curvature and length, and is inversely proportional to viscosity.^21,22^ With the viscosity of the liquid remaining constant throughout, the geometry of the blood vessel can have a predominant effect on flow profile.^23^ Therefore, constant branching and associated reduction in diameter could alter the flow profile of plasma. Turbulence is a crucial feature observed in plasma flow that arises due to changes in vessel physiology.^24^ Such changes in plasma flow caused by vascular dynamics could potentially affect the distribution of proteins in the blood and their interactions with NPs.^25^ A classic example of how vessel structure affects the flow and its implications is the increased probability of plaque depositions and leukocyte adhesion at vessel bifurcations.^26,27^ Unfortunately, most studies only evaluate flow rate and do not consider vessel architecture.

This study analyses NP protein interactions in the presence of a single bifurcation with a decrease in the diameter of the daughter vessels to determine the impact on the composition of the PC that forms on polystyrene NPs. The results show that the changes in vessel structure indeed influence the amount and composition of the PC on NPs. Changes in the NP uptake were also observed due to differences in the PC composition. These results have implications for the ability to translate *in vitro* PC studies *in vivo* and suggest the need to include the complexity of the vasculature in the design of *in vitro* studies.

## Results and discussion

### Modeling the vasculature *in vitro* to study effort on protein corona formation

**Fig. 1:**
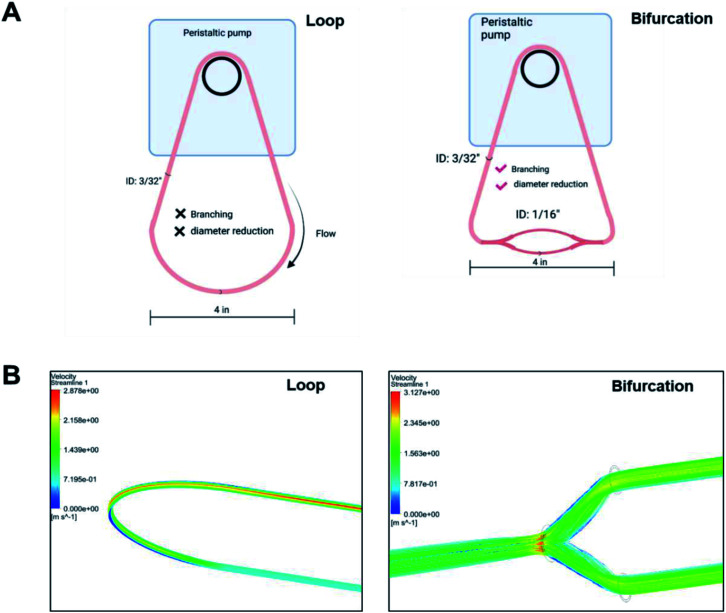
(A) Scheme of *in vitro* models to ascertain effect of bifurcations on the PC formed on NPs (B) ANSYS fluid modeling of plasma flow in loop and bifurcated *in vitro* systems.

Blood vessels branch into smaller daughter vessels that change blood flow velocity. Considering that the flow rate of plasma affects the formation of PC on NPs, we hypothesized that changes in hemodynamics caused by bifurcations and the associated reduction in blood vessel diameter impact the composition of the PC formed on an NP. *In vitro* models made from polyurethane tubing connected to a peristaltic pump to form a closed-loop circuit or incorporating bifurcations (Fig. 1A) were created to test the hypothesis. For the bifurcation model, a central tubing with 3/32 inches internal diameter (ID) branched into two daughter tubes of 1/16 inch ID at an angle of 70° between them (Fig. 1A). Fluid dynamics were modeled for these vessel architectures with ANSYS Fluent software to evaluate how hemodynamics are affected. The modeling of fluid flow in the loop system had a maximum velocity of 2.9 m s^−1^ at the center that deceased significantly closer to the vessel walls. This is expected as flow in cylindrical tubes has maximum velocity in the center, while the velocity decreases closer to wall due to boundary condition.^28^ While there is no fluid mixing in the simple loop, the addition of the bifurcations increased mixing, causing turbulence at the branch junction. In the models with bifurcation, fluid velocities peaked at 3.1 m s^−1^ at the branch point, 6% faster than the highest flow rate predicted in the loop configuration. Immediately after the branch point, the fluid velocity drastically reduced within the arms of the branches (1.6 m s^−1^) before increasing to a maximum of 2.3 m s^−1^ at the center of the daughter vessels. These *in silico* modeling results show the effects that the dramatic changes in the vessel architecture could have on fluid flow and corresponds to flow rate variations modeled for *in vivo* bifurcations.^29^

### Bifurcation affects the amount of proteins on NPs

**Fig. 2:**
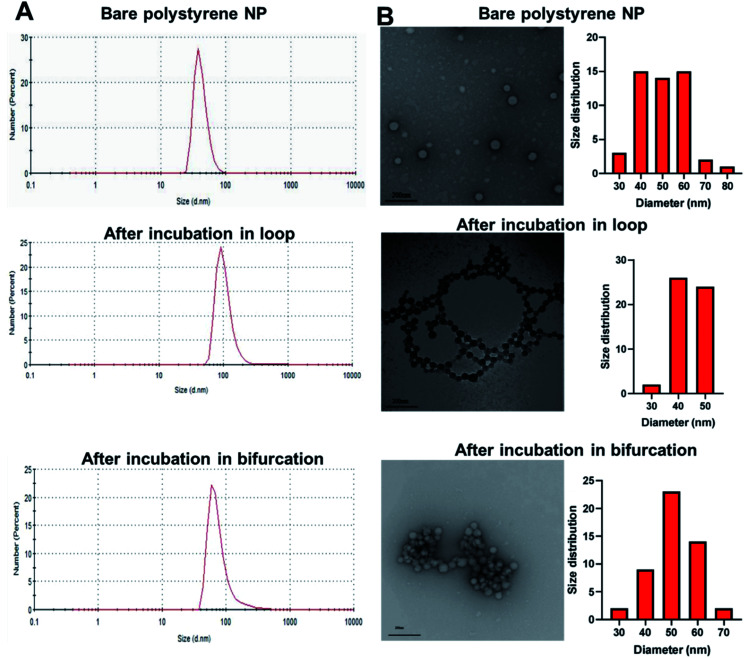
(A) Hydrodynamic diameter of polystyrene nanoparticles (PS-NPs) before and after incubation in loop and bifurcation flow systems containing plasma. (B) Transmission electron micrograph of bare PS-NPs, PS-NPs incubated in loop system and PS-NPs incubated in bifurcation system.

To investigate the potential effect of bifurcation on the protein corona, 50 nm amine-modified polystyrene NPs were purchased and used without any modifications. Polystyrene NPs (PS-NP) are used as a model NP in these studies because their uniformity in size (polydispersity index = 0.06) would reduce possible confounding results due to size distributions. The hydrodynamic size of the PS-NPs was measured as 54 ± 1 nm, while the core size, derived from transmission electron microscopy (TEM), was 50 ± 10 nm (Fig. 2 and Table 1). The PS-NPs were injected into circulating plasma in the loop and bifurcation systems at a flow rate of 25 mL min^−1^ for 10 minutes. The particles were washed thrice with 1x PBS to remove the loosely bound proteins (soft PC), leaving behind proteins tightly bound to the surface called the hard PC. The resultant NP-hard PC complex was used for all the further analyses. The hydrodynamic diameter of the PS-NPs incubated in the loop system was 66 ± 27 nm, demonstrating an increase in the hydration shell due to the adsorbed hard PC. The TEM size remained unchanged (Fig. 2 and Table 1). After incubation of the PS-NPs in the bifurcation model, the hydrodynamic diameter increased to 56 ± 18 nm, while the TEM measured size was 51 ± 8 nm (Fig. 2 and Table 1). The zeta (*ζ*) potential of the untreated amine-modified PS-NPs was +42 ± 5 mV and changed to −22 ± 4 mV and −25 ± 2.4 mV after incubation in the plasma in the loop and bifurcation systems, respectively (Table 1).

**Table 1:** Particle characteristics

Particle	Hydrodynamic diameter (nm)	Zeta potential (mV)	PDI	TEM (nm)
Nanoparticle only	54 ± 1.2	42 ± 5.2	0.06	50 ± 10
Loop PC	66 ± 27.0	−22 ± 4.0	0.5 ± 0.1	44 ± 5
Bifurcation PC	56 ± 18.0	−25 ± 2.4	0.4	51 ± 8

The plasma proteins bind to the surface of NPs upon immediate contact with its surface, reducing the exposure of amines on the NP surface that are responsible for the cationic charge. The *ζ* potential reversal of the NPs upon exposure to plasma indicates the preferential binding of proteins with low isoelectric points (pI) to the cationic NP surface. Albumin, the most abundant protein in blood plasma and generally present in the PC has a pI of 4.9.^30,31^ Similarly, complement proteins and apolipoproteins, which generally form part of the PC, have low pIs.^31^ Although the TEM micrograph of the untreated PS-NP showed individual unaggregated particles, PS-NPs from the loop and bifurcation treatment showed the tendency to aggregate. However, the pattern for aggregation was different for both treatments. The PS-NP from the loop treatment aggregated in series to form chain-like structures (Fig. 2), while the PS-NPs from the bifurcation treatment aggregated into large clusters. The amount of protein on NPs influence how they aggregate.^32,33^ At optimal concentrations, proteins improve the distribution of NPs and prevent aggregation. However, higher concentrations of proteins on NPs tend to increase particle aggregation.^33^ Proteins that bind to NP surfaces undergo conformation changes and could induce protein aggregation, enhanced by the increased turbulence in the bifurcation model. This can ultimately lead to coalescence of the protein-coated NPs into aggregates. These results suggest that different amounts of proteins with different compositions adsorb to the NPs after treatment under the two conditions. This is anticipated as the flow rate of plasma has been shown to affect the amount of proteins on NPs.^17^

**Fig. 3:**
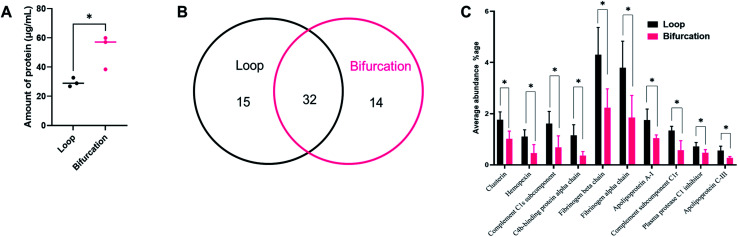
(A) Micro-BCA assay to quantify proteins in the corona formed on NPs after loop and bifurcation treatment. (B) Venn diagram showing the common and different proteins between loop and bifurcation treatment as identified by LC-MS/MS. (C) Comparison between common proteins identified in loop and bifurcation treatment (only statistically significant proteins are shown here).

We confirmed that the change in flow velocity caused by the introduction of the bifurcation impacted the amount of proteins in the hard corona by micro-BCA assay. The addition of the bifurcation increased the amount of protein on the PS-NPs by 75% compared to the loop system (Fig. 3A; *p* = 0.0324). These results are supported by other studies that show that NPs isolated after *in vivo* injection have more adsorbed proteins than *in vitro* incubated NPs, supporting a role for the vascular architecture.^15,34^ We next determined whether the difference in the amount of protein in the PC also translated into variations in its composition using liquid chromatography-tandem mass spectrometry (LC-MS/MS). Four independent experiments were performed for each condition, and proteins with an abundance greater than 0.2% were analyzed. The protein had to be present in at least three experiments for it to be considered for further analysis.

Of the most abundant proteins identified by LC-MS/MS in the hard corona, 15 unique proteins were present only in the corona for NPs injected in the loop system. In comparison, 14 unique proteins were present in only the PC from the NPs injected into the bifurcation system. There were 32 proteins present in the PC from NPs treated in both the loop and bifurcation systems.

Although 32 proteins were shared between the two models, their abundances varied and were impacted by the flow models (Fig. 3B). Fig. 3C represents the 10 proteins that showed significant differences in abundance between the two conditions. While overall, bifurcation increased the amount of proteins in the PC, the shared proteins were most abundant in the PC from the loop condition (Fig. 3C).

### Bifurcation changes the composition of the protein corona on PS-NPs

**Fig. 4:**
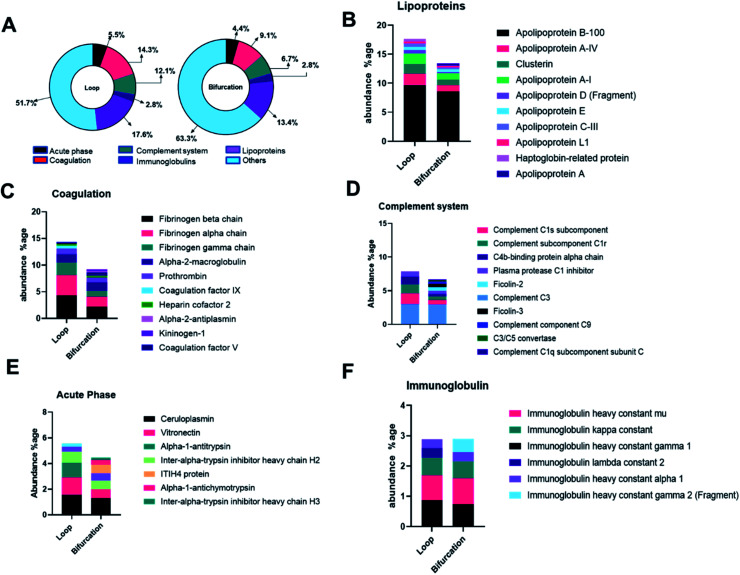
(A) Donut chart depicting the proteins classified according to the biological processes. Annotation of proteins in the protein corona into acute phase reactants (B), coagulation related proteins (C), immunoglobulin (D), complement system protein (E) and lipoproteins (F).

The proteins were categorized depending on their contribution to acute phase reactions, coagulation, complement response, immunoglobulins, and lipoproteins. These protein groups were selected because of their contribution to NP biodistribution, pharmacokinetics, clearance, and cellular interactions.^19,34^ For example, lipoproteins, acute phase reactants, immunoglobulins, and the exclusion of coagulation and complement proteins in the PC of injected NPs increase their blood residence time.^34^ 48.3% and 36.7% of the LC-MS identified proteins fell in the categorized groups for loop and bifurcation treatments, respectively (Fig. 4A). Of these proteins, lipoproteins were most abundant, representing 18% and 13% in abundance in the PC on NPs from the loop and bifurcation flow systems, respectively. Apolipoprotein (Apo) B-100 was the most abundant lipoprotein accounting for more than 50% of the lipoprotein content in PC of NPs from both loop and bifurcation models. While present in both PCs, clusterin, Apo A-I and Apo C-III demonstrated statistically significant differences in abundance (Fig. 4B and 3C). Proteins associated with coagulation were most prominent after lipoproteins and showed higher abundance in the PC of NPs from the loop system. The abundance of fibrinogen components (alpha, beta, and gamma chains) was twice in the PC of NPs from the loop system compared to the PC from the bifurcation system. The PC from the loop also lacked alpha-2-antiplasmin and kininogen-1, which were present in PCs from bifurcation, albeit at low abundance. The composition of complement system-related proteins in the PC of NPs from the bifurcation system was more diverse, with 10 different proteins compared to just 5 in the PC from the loop system. Similarly, there were differences in the identity of proteins related to acute phase reactions and immunoglobulins (Fig. 4B–F).

**Fig. 5:**
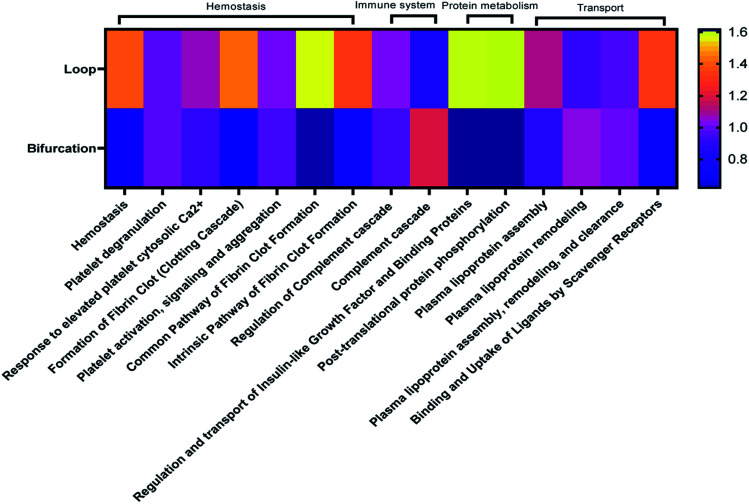
Heatmap showing top 20 biological pathways predominant in identified proteins.

Considering that the introduction of bifurcations affected the PC composition on the NPs, the potential impact on biological pathways was investigated. For this, the annotated LC-MS/MS data were analyzed using Reactome Pathway Database, a free, open-source bioinformatics tool that maps pathways by analyzing the over-representation of proteins associated with specific pathways in a given data set.^35–37^ The analyses show enrichment of proteins associated with different pathways in the PC on the NPs (Fig. 5). Although there was equal enrichment of proteins associated with platelet activation, aggregation, and degranulation in the PC of NPs from both flow systems, proteins associated with fibrin clot formation were more enriched in the PC from the loop. These together resulted in pathways associated with hemostasis being enriched in the PC on NPs from the loop system. Pathways associated with complement activation and progression were more abundant in the PC from the NPs that flowed through the bifurcation model. However, pathways contributing to insulin-like growth factor protein transport were enriched in the loop model.

### The PC formed on NP due to bifurcation disrupt cellular uptake

**Fig. 6:**
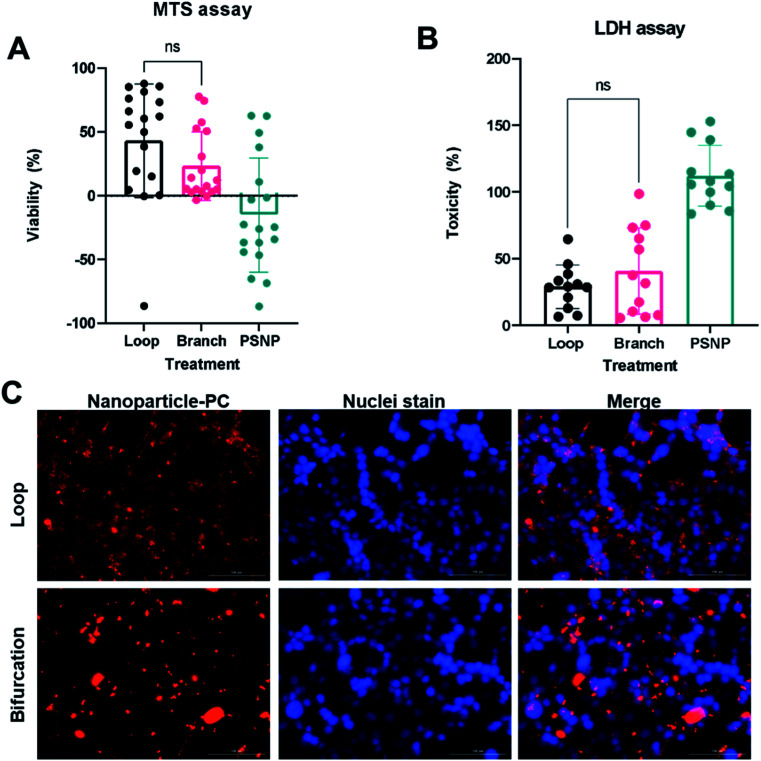
Interactions of nanoparticle-protein corona (NP-PC) complex with cells. Toxicity of NP-PC to 4T1 cells assessed by MTS assay (A) and LDH assay (B). (C) Fluorescent images of NP-PC uptake by 4T1 cells.

The PC mediates NP cellular interaction and uptake endocytosis.^38^ The PC-coated nanoparticles (PC-NPs) were incubated with 4T1 breast cancer cells to assess cellular uptake and toxicity. Both MTS metabolic assay and lactate dehydrogenase (LDH) viability assay show that bare PS-NPs are toxic to the 4T1 cells after incubation for 24 hours in serum-free conditions (Fig. 6A and B). The presence of the protein corona reduced NP-induced toxicity, which was independent of how the PC was formed (Fig. 6A and B). The toxicity of many NPs is mediated by their surface chemistry.^39–41^ Cationic NPs such as these PS-NPs induce toxicity by disrupting the cell membrane, with their toxicity rising with increasing *ζ*-potential.^40,42^ The PC on the NPs masks this cationic surface and prevents direct interaction with the cell membrane.

The PC-coated NPs (PC-NP) were incubated with 4T1 cells for 4 hours. The uptake studies were performed in serum-free media to ensure that any observed effect is from the proteins associated with the NPs. The NPs with PC from the loop system showed uptake by the 4T1 cells, which were distributed throughout the cytoplasm, and did not show a preference for perinuclear localization (Fig. 6C). However, the NPs with PC generated in the bifurcation flow system showed minimal uptake by the 4T1 cells. The NPs mainly remained outside the cell, associated with the cell membrane as clusters (Fig. 6C). Our initial TEM analysis of the PC-NPs showed that NPs incubated in the bifurcation system tended to form clusters (Fig. 2B), likely due to the greater amount of proteins in the PC. The increased hydrodynamic shear forces experienced by NPs and proteins in the bifurcation model could also cause protein unfolding and denaturation that increase aggregation. The cellular uptake of NPs is a function of their size, with NPs larger than 100 nm showing lower uptake.^42,43^ Although the individual NPs are 50 nm and readily endocytosed, their aggregated size of greater than 500 nm decreases their endocytosis by the cells. While this aggregation may hinder their uptake by 4T1s, which are of epithelial origin, they may increase uptake by macrophages that remove debris.^44,45^ These results suggest the probability that the NPs would aggregate and accumulate in the liver and spleen, lowering their blood residence time *in vivo*.

### The protein corona is impacted by both branching and size reduction

**Fig. 7:**
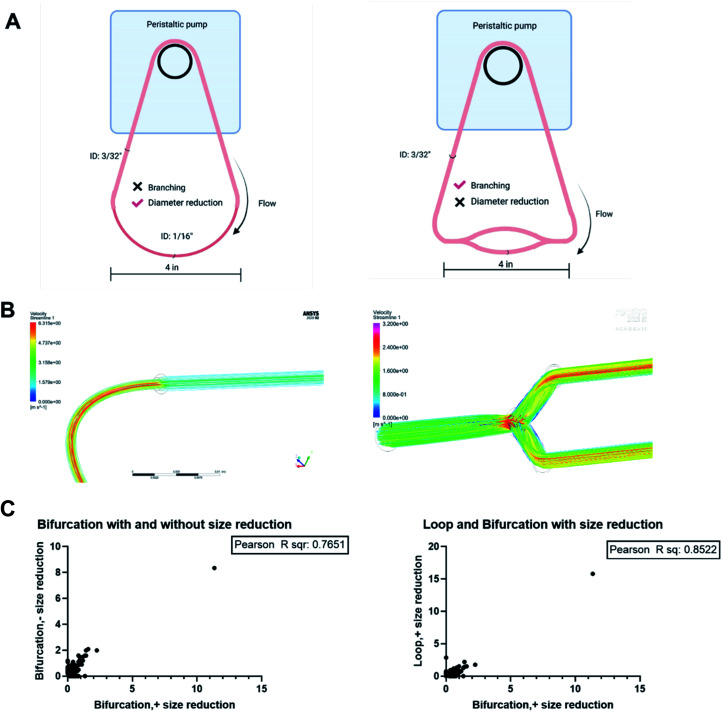
(A) *In vitro* vascular models. (B) Flow simulation through different architecture to observe the differences in flow velocity. (C) Correlation analyses to determine impact of bifurcation and size reduction on the PC formed on NPs.

The results obtained showed that bifurcation indeed affects the PC formed on NPs. However, they raise the question of what component of the bifurcation, the branching, reduction in diameter of the daughter vessels, or combination of both is responsible for the observed changes. The PC formed on the PS-NPs after flow in two other flow systems; a bifurcation model without the decrease in the diameter of daughter vessels and a loop system that incorporates a decrease in size were characterized to determine this (Fig. 7A). Dynamic flow simulations show that decreasing the diameter of the vessel increased fluid velocity significantly, reaching speeds of 6.3 m s^−1^ at the center of the vessel. In the branching model without size reduction, in the portion of daughter vessels, there is an increase in velocity at the branch point that is followed by a decrease within the branch. However, the decrease in velocity is not as dramatic as observed in the standard bifurcation model with a decrease in the diameter of daughter vessels. The composition and amounts of proteins, as abundance percentage, in the PC on NPs from these two models were compared to those from the standard bifurcation model (Fig. 7C). Comparison of PC of NPs from bifurcation with size reduction to those from bifurcation without size reduction in daughter vessels yielded an *R*^2^ = 0.76. Comparison of the PC from the bifurcation with size reduction to those from the loop with size reduction produced an *R*^2^ = 0.85. These results demonstrate that the PC composition on NPs results from a combination of the branching and reduction in the size of the daughter vessels.

## Conclusion

The study presented here demonstrates the impact of bifurcation, a common feature of blood vessels on the PC formed by NPs. Bifurcations increase the amount of and change the composition of proteins adsorbed to NPs and ultimately determine their interaction with cells. This is the first study to explore how different aspects of blood vasculature could contribute to the formation of the PC on injected NPs. Considering the current discordance between PC formed on NPs *in vitro* and *in vivo*, this study suggests the varied results may be partly due to the vessel structure and its impact on fluid dynamics. Therefore, there is a need to investigate better how vascular characteristics impact the PC to understand their formation *in vivo* better and create better prediction models.

## Materials and methods

### Materials

50 nm blue fluorescent amine-modified polystyrene NPs were purchased from Millipore Sigma (Burlington, MA) and were used without further modification. Human plasma with K2 EDTA anticoagulant was purchased from Innovative Research (Novi, MI) and centrifuged to remove aggregated protein mass. Polyurethane tubing and fittings of desired sizing and shape were purchased from United States Plastic Corps (Lima, OH).

### Protein corona formation on nanoparticles

Flow conditions for plasma proteins were established using a peristaltic pump. Polyurethane tubing of desired geometry was constructed using appropriate fittings and connected to the pump. The bifurcation model was created using polypropylene fitting with a 70° angle between the two arms. To mimic an artery with branching followed by a reduction in diameter, polyurethane tubing with an inner diameter of 3/32 inner diameter (ID) was attached to two smaller 4 inches diameter tubing of 1/16 inches ID *via* the “Y” fitting. The smaller tubing ended in another “Y” fitting connected to a larger diameter tubing to create a closed circuit vessel model. The simple loop model was constructed using silicone tubing of the equivalent length of the bifurcation circuit. The volume of plasma in both systems remained equal at 2 mL.

The models were filled with 100% human plasma (2 mL) and set to a 25 mL min^−1^ flow rate using a peristaltic pump (Fisherbrand™ Variable-Flow Peristaltic Pumps). Using an insulin syringe, polystyrene NP(1 mg mL^−1^) was injected in the plasma flow direction and allowed to circulate for 10 minutes at 37 °C. After 10 minutes, the NPs were recovered from the plasma by centrifugation (Thermo Scientific™ Sorvall™ Legend™ Micro 21 Microcentrifuge) at 21000 g for 30 minutes. The recovered NPs were washed in phosphate-buffered saline (PBS) three times to remove the soft corona, with centrifugation recovery after each wash.

### Particle characterization

Transmission electron micrography (TEM) (JEOL 1200EX) confirmed the size distribution and morphology of plasma-treated and untreated nanoparticles. Nanoparticles were drop-cast onto copper grids, stained with uranyl acetate, and air-dried at room temperature before imaging.

Hydrodynamic diameter, polydispersity index, and zeta potential measurements were performed on a Malvern Zetasizer-Nano instrument on particles resuspended in ultrapure water at room temperature.

The amount of protein on NP pellets after running through the flow models was obtained after washing three times with 1x PBS by micro-BCA assay (ThermoFisher, Waltham, MA) following the manufacturer's instructions.

### Mass-spectrometry

#### Sample preparation

Protein digestion was carried out by S-Trap sample processing technology (S-TRAP™) according to the protocol. Briefly, reductant (1 μL) was added to protein (5 μg) and was dissolved in 1X lysis buffer. After incubation for 15 minutes at 55 °C, an alkylator (1 μL) was added, and the solution was incubated for 10 minutes. The pH of the solution was adjusted to 1 with acid and digested overnight at 37 °C. The peptides were eluted using elution buffer, dried, and resuspended to the desired LC-MS/MS analysis volume.

### LC-MS/MS

LC-MS/MS analysis used a Thermo Scientific UltiMate 3000 nanoUHPLC system coupled to an Orbitrap Fusion mass spectrometer. Dried samples were reconstituted in a mixture containing 98% water, 2% acetonitrile, and 0.1% formic acid (20 μL) and the injection volume was set to 1 μL. Samples were separated with an Acclaim PepMap C18 column (0.075 × 150 mm, 2 Å particle size) at a flow rate of 0.400 μL min^−1^. The mobile phase consisted of water, acetonitrile, and formic acid (98/2/0.1 for buffer A, 2/98/0.1 for buffer B). The gradient was as follows: equilibration at 2% B for 5 minutes, ramping up to 45% B at 37 minutes, 90% B at 40 to 46 minutes, followed by re-equilibration at 2% B at 47 to 60 minutes.

Eluted peptides were introduced into the mass spectrometer by positive ESI at 2450 V, and the ion transfer tube temperature was set to 275 °C. Data was obtained in top speed mode with a cycle duration of 3 seconds. Full scans were acquired in the Orbitrap at a resolution of 120 000 at *m*/*z* 200. The mass range, RF lens amplitude, and maximum injection time were 400–1600 *m*/*z*, 60%, and 100 ms, respectively. MS/MS spectra were acquired in the ion trap set to a rapid scan rate. The precursor isolation window was set to 1.6 *m*/*z*. Fragmentation was achieved by HCD at a fixed collision energy of 28%. Dynamic exclusion was set to 60 seconds with a tolerance of 10 ppm.

Data were processed by use of Thermo Scientific Proteome Discoverer 2.4. A custom theoretical fragment database was derived from the human reference proteome available from UniProt (UP000005640, 78120 protein sequences). Spectra were processed and matched against the database using the basic SequestHT processing and consensus workflows included with the software and tailored for the tribrid platform, with the precursor and fragment tolerances set to 5 ppm and 0.6 Da, respectively. Two peptides were required for the successful identification of a protein. The abundance percentage of each protein was calculated by dividing each peptide number by the sum of peptides in that particular group. The abundance percentage thus calculated was used for all the analyses.

### Cell culture

4T1 breast cancer cell lines were purchased from the American Type Culture Collection (ATCC, Manassas, VA). The cells were grown and maintained in RPMI supplemented with 10% fetal bovine serum (v/v), 1% penicillin–streptomycin in a 5% CO_2_ incubator at 37 °C.

The 4T1 cell lines were passaged at 70–80% confluency and were seeded in a 96 well-plate, with each well comprising 10 000 cells. The cells were allowed to attach to the well-plate prior to administration with the nanoparticle.

### Viability assay

Cell viability was assessed 24 hours after incubation with bare NPs or NPs with PC using the Lactate Dehydrogenase assay (CytoTox 96® Non-Radioactive Cytotoxicity Assay) and MTS assay (CellTiter 96® AQueous Non-Radioactive Cell Proliferation Assay) kit, following manufacturer's protocols.

### Cell uptake of NPs

After incubation in plasma under the different flow conditions and washing in PBS to remove the soft corona, the PC coated PS-NPs were fluorescently labeled with Alexa Fluor 594 NHS ester (ThermoFisher Scientific, Waltham, MA). The NPs were sonicated for 5 minutes in a bath sonicator (VWR® Ultrasonic Cleaners) to break down the aggregates before addition to cells.

The protein corona coated NPs (25 μg mL^−1^) were incubated with 4T1 cells at a seeding density of 10 000 cells per well in a 96 well-plate, in serum-free media for 4 hours. The cells were washed three times with phosphate-buffered saline (PBS), stained with Hoechst 33258 dye, and fixed in 4% paraformaldehyde before imaging.

### Cell imaging

The 4T1 cells were imaged using a Lionheart LX automated microscope (Biotek, Winooski, VT) using the filter cubes corresponding to Texas red channel to image NPs and DAPI channel to image cell nuclei.

### Statistical analysis

GraphPad PRISM version 9.1 (La Jolla, CA) was used to perform statistical analyses with the error bars denoting standard deviation. Statistical comparison was determined by Student's *t*-test and the outcome are denoted as follows: *: *p* < 0.05; **: *p* < 0.01; ***: *p* < 0.0001; ns: not significant.

### Correlation analysis

Each treatment was regarded to be an independent variable. Pearson correlation was performed on abundance percentages of proteins from each treatment and graphed using GraphPAD PRISM version 9.1 (La Jolla, CA).

## Author contributions

Sridevi B. Conjeevaram – data curation, formal analysis, investigation, validation, visualization, and writing original draft and editing. Isaac M. Adjei – conceptualization, funding acquisition, resources, supervision, writing – original draft, review, and editing. Ryan M. Blanchard: methodology, manuscript review. Amulya Kadaba: formal analysis, manuscript review.

## Conflicts of interest

The authors declare no conflict of interest.

## Supplementary Material
